# Engineering Pathways in Central Carbon Metabolism Help to Increase Glycan Production and Improve *N*-Type Glycosylation of Recombinant Proteins in *E. coli*

**DOI:** 10.3390/bioengineering6010027

**Published:** 2019-03-21

**Authors:** Benjamin Strutton, Stephen RP Jaffe, Caroline A Evans, Gregory JS Fowler, Paul D Dobson, Jagroop Pandhal, Phillip C Wright

**Affiliations:** 1 Department of Chemical and Biological Engineering, The University of Sheffield, Sheffield S1 3JD, UK; bstrut@hotmail.co.uk (B.S.); s.jaffe@sheffield.ac.uk (S.R.P.J.); Caroline.Evans@sheffield.ac.uk (C.A.E.); g.fowler@sheffield.ac.uk (G.J.S.F.); scruffy.biotech@gmail.com (P.D.D.); 2 Scruffy Biotech Ltd. Green Bank, Derbyshire SK13 6XT, UK; 3 School of Engineering, Faculty of Science, Agriculture and Engineering, Newcastle University, Newcastle Upon Tyne NE1 7RU, UK; Phillip.Wright@newcastle.ac.uk

**Keywords:** *N*-glycosylation, *Escherichia coli*, glycosylation efficiency, cell engineering, recombinant protein production

## Abstract

*Escherichia coli* strains have been modified in a variety of ways to enhance the production of different recombinant proteins, targeting membrane protein expression, proteins with disulphide bonds, and more recently, proteins which require *N*-linked glycosylation. The addition of glycans to proteins remains a relatively inefficient process and here we aimed to combine genetic modifications within central carbon metabolic pathways in order to increase glycan precursor pools, prior to transfer onto polypeptide backbones. Using a lectin screen that detects cell surface representation of glycans, together with Western blot analyses using an *O*-antigen ligase mutant strain, the enhanced uptake and phosphorylation of sugars (*ptsA*) from the media combined with conservation of carbon through the glyoxylate shunt (*icl*) improved glycosylation efficiency of a bacterial protein AcrA by 69% and over 100% in an engineered human protein IFN-α2b. Unexpectedly, overexpression of a gene involved in the production of DXP from pyruvate (*dxs*), which was previously seen to have a positive impact on glycosylation, was detrimental to process efficiency and the possible reasons for this are discussed.

## 1. Introduction

Glycosylation is regarded as one of the most common post-translational modifications of proteins, occurring on almost half of all proteins in nature [1]. The addition of a sugar group impacts on protein biophysical properties, which can influence native functions, for example, activity, localisation and half-life. Although present across all domains of life, eukaryotic systems remain the most characterised and therefore the best understood. In humans, it is thought that nearly half of all proteins are modified by the addition of sugars, with 90% of the molecular mass of some glycoproteins made up by glycans. More recently, a growing interest in prokaryotic glycosylation has led to improved understanding of the molecular mechanisms involved and the functional traits associated with this relatively energy-expensive protein modification process. However, overall knowledge is relatively poor due to the diversity of glycan structures and variability of the processes in prokaryotic systems. 

*Escherichia coli* is a well-established protein production cell factory, which has been exploited industrially for several decades. From high value therapeutics to high bulk industrial enzymes, *E. coli* strains have been modified and refined to create an array of industrial host cell platforms. This is largely due to its fast growth, cheap cultivation requirements and its easy-to-manipulate genome. Moreover, the community has been rewarded by its success, meaning that our understanding of the genetic complexity within strains has improved as it has been widely used as a model system. Although *E. coli* is used for the production of a variety of proteins, it can struggle to express certain types. These are often larger, complex proteins, where multiple transmembrane helices might be present, making expression difficult. There can also be a need for modifications, for example, disulphide bridges. Many alterations have been made to the *E. coli* cell chassis to improve recombinant protein production to overcome these expression challenges. For example, the well-studied “Walker strains” C41 (DE3) and C43 (DE3) were generated, which enable relatively high levels of membrane protein expression [2]. The mutated cells accumulate extra membrane space by mutations in their *lac*UV5 promoter. Similarly, strains such as CyDisCo or SHuffle are able to express proteins which require bonds between cysteine groups on amino acid side chains, requiring a reducing environment. CyDisCo cells specifically utilise the expression of the sulfhydryl oxidase Erv1p and a disulphide isomerase (PDI) [3,4] to enable efficient bond formation in the cytoplasm, instead of relying on transport to the periplasmic space [5,6]. These strains have since been commercialised. 

*N*-glycosylation involves the addition of glycan structures to the asparagine side chain in polypeptide structures in cells. This cellular process has been characterised in several Gram-negative bacteria, mechanistically as well as functionally [7,8]. The functional transfer of the *Campylobacter jejuni* protein glycosylation pathway into *E. coli* predictably led to excitement amongst the recombinant protein community [9], where *E. coli* could be used to produce glycoproteins, whether for diagnostics, therapeutics, or simply as a research tool to better understand the functional attributes of specific glycoforms. Molecular engineering efforts have demonstrated that *E. coli* can glycosylate different proteins, from bacterial to eukaryotic origin [9,10,11]. Moreover, the sugar structures can be changed, and the core eukaryotic trimannosyl-chitobiose has been constructed by incorporating different genes into cells [12,13]. 

Despite these ambitious efforts to create new *N*-linked glyco-profiles in *E. coli* by exploiting the *C. jejuni* cellular machinery, the productivity of the system remains relatively poor. Investigations into how the protein structure affects enzyme access to the *N*-glycosylation consensus sequence have revealed optimal surrounding amino acids [14]. Process optimisations include improved gene expression induction regimes and media formulations [15]. Although proteins are synthesised in a template-driven process as part of the central dogma of molecular biology, glycans are not. They are secondary gene products, which are constructed through a series of metabolic steps controlled by proteins (mostly enzymes) [16]. This is an energetically demanding process and, therefore, *E. coli* suffers from a degree of metabolic burden [17]. Efforts to engineer the cells to overcome these constraints have been systematic, based on the existing body of literature pointing to likely gene targets for manipulation [18,19], employed omics-based metabolic engineering [20] or inverse metabolic engineering [10], to highlight pathways that can be tested to improve production. These latter approaches have successfully improved the total amount of protein carrying the desired glycan structure, without a significant loss of total recombinant protein yield. Isocitrate lyase (IcL) is a key enzyme in the glyoxylate pathway that cleaves isocitrate to succinate and glyoxylate. Through its overexpression in *E. coli*, a three-fold increase was observed in glycosylation efficiency [20]. It was theorised that utilisation of the glyoxylate pathway instead of the full citric acid cycle conserves carbons, and these excess carbons could be used in the production of glycan precursors. Similarly, the *ptsA* gene encoding for the phosphoenol-pyruvate (PEP) protein phosphotransferase system enzyme I was overexpressed in *E. coli* cells, resulting in almost a seven-fold increase in the production of glycoprotein AcrA [10]. PtsA is initially phosphorylated by PEP and subsequently transfers its newly acquired phosphoryl group to a histidine phosphocarrier protein which in turn continues the cascade [21,22]. The pathway was discovered in *E. coli* and found to be involved in the uptake and phosphorylation of a variety of sugars, including *N*-acetylglucosamine, which is potentially beneficial to the bacterial glycosylation process. In the same study, overexpression of *dxs* gave a 1.3-fold increase in glycosylation efficiency. The *dxs* gene encodes for 1-deoxyxylulose-5-phosphate (DXP) synthase, which catalyses the production of DXP from pyruvate and D-glyceraldehyde-3-phosphate (D-GAP) in a thiamine diphosphate-dependent manner [23,24]. The production of DXP is deemed to be the rate-limiting step in the mevalonate pathway of isoprenoid synthesis [25], but downstream of DXP synthesis, it is involved in the production of a number of molecules including undecaprenyl phosphate (Und-P), which acts as the bacterial lipid anchor on which the glycans are built. These modifications target metabolic processes where carbon flux can be diverted from central carbon metabolism and re-directed towards the recombinant glycosylation pathway, or enable efficient conversion of carbon without compromising cellular growth rates.

In this study, the effect of the overexpression of these three genes (*ptsA, icL and dxS)*, in various combinations, were analysed using a lectin screen that detects cell surface representation of glycans through the exploitation of the native *E. coli O*-antigen ligase, WaaL, in MC4100 *E. coli* cells. Western blot analysis was conducted to determine the protein glycosylation efficiency and relative glycoprotein production in the bacterial strain, CLM24, where *waaL* has been knocked out to prevent cell surface representation of recombinant glycans [26]. The aim was to firstly characterise the effect of the overexpression of these previously identified genes on glycan production, followed by impacts on glycosylation efficiency. The bacterial exemplar *N*-glycoprotein, AcrA, was initially used as it is the most understood target glycoprotein, together with the model bacterial N-glycosylation machinery termed protein glycosylation pathway 2 (pgl2) [27]. To explore whether the engineered cells could improve the glycosylation of an industrially relevant protein, IFN-α2b was also tested as a target glycoprotein. IFN-α2b is a frequently used cytokine for therapeutic purposes and commercial variants are produced in *E. coli* e.g., Grippferon^®^ (Firn-M, Moscow, Russia) [28]. It contains a known *O*-glycosylation site in a relatively unstructured region, and this was modified to enable bacterial *N*-glycosylation. 

## 2. Materials and Methods

All materials were purchased from Sigma-Aldrich (Dorset, UK), with working antibiotic concentrations of 100 µg/mL ampicillin, 35 µg/mL chloramphenicol, and 35 µg/mL kanamycin used where appropriate, unless stated otherwise.

### 2.1. PCR, DNA Cloning and Vectors

The strategy employed to overexpress the genes of interest was to place them under a constitutive promoter on the same plasmid as the target protein. To achieve this, a new multiple cloning site (MCS) was inserted into pEC(acrA). The MCS contained the necessary restriction sites to insert the three genes of interest and was designed with the constitutive promoter, J23119, from the iGEM catalogue (Table S1). This 35 bp promoter was placed upstream of the restriction sites required for insertion. Forward primers used to amplify the genes of interest contained the Shine Dalgarno sequence, AGGAGG, and a six base pair gap to ensure the ribosome binding site (RBS) was situated upstream of the start codon (Table S2). Upon creation of the various constructs, plasmids were sent off for sequencing to confirm insertion and to ensure that no PCR errors were incorporated. 

PCR reactions: 98 °C for 3 min, 30 cycles of 30 s at 98 °C, 30 s at the annealing temperature of the primer, and an extension step at 72 °C run at 1 kb per 30 s, extended depending on the size of the fragment being amplified. A final extension step was also run at 72 °C for 10 min before cooling the reaction to 4 °C. 

Post amplification of the MCS, the reaction was subjected to agarose gel analysis, with the DNA being extracted using a Qiagen^®^ kit (Hilden, Germany) prior to digestion with EcoRI and XmaI at 37 °C for 1 h. The target plasmid pEC (acrA) was also digested before insertion of the MCS using T4 ligase (NEB) following the manufacturer’s protocol to create the pEC (acrA_MCS) plasmid. The three genes to be inserted were amplified from the plasmids outlined in the aforementioned studies [10,20] and cloned into pEC (acrA_MCS) with the specific restriction enzymes that were required for the particular inserts. The seven constructs were all validated with restriction digestion and sequence analysis prior to transformations in the appropriate strains with pACYC (pgl2).

The amino acid sequence for an IFN-α2b with a *C. jejuni* glycosylation consensus sequence was designed by locating a suitable region based on the crystal structure available in the PDB (1ITF). The T corresponds to residue T106, an *O*-linked glycosylation site and due to being in a relatively unstructured region on the protein, it was chosen as the site for modification. A glutamic acid (E) and asparagine (N) were inserted at site 102 and 104, respectively, into the amino acid sequence using the nucleotide sequence for IFN-α2b (NCBI sequence ID CAA23809.1), together with the pelB signal sequence and a C-terminal hexa-his tag sequence (Supplementary Files). The subsequent sequence was codon optimised, synthesised by DNA 2.0 and used to replace *acrA* in the pEC (acrA_MCS) plasmid. A Western blot using a lectin peroxidase blot was used to test for glycosylation as described previously [10].

### 2.2. Cell Surface Representation of Glycans

One mL of LB in a sterile 1.5 mL centrifuge tube was inoculated with the bacterial strain of interest, and the appropriate antibiotics were added. Oxygen-limited growth took place overnight at 37 °C shaking at 180 rpm to limit cell density. The O.D at 600 nm was measured and cultures were normalised down to 0.6 using sterile deionised water. The cells were then diluted by a factor of 1 in 75,000 using sterile deionised water to a final volume of 1 mL. One-hundred µL of the diluted cells were plated out on LB agar with the appropriate antibiotics and left to incubate and grow aerobically at 37 °C overnight. A piece of Protran™ nitrocellulose paper was cut to fit a petri dish and soaked in the appropriate antibiotics for 5 min before being left to dry in a flow hood. The paper was placed over the petri dish, making contact with the colonies and left to incubate for 3 h at 37 °C. The nitrocellulose membrane was blocked in PBS containing 2% Tween 20 for 2 min at room temperature, before being washed twice with PBS for 10 min. The membrane was incubated in PBS with 0.05% TWEEN^®^ 20, 1 mM CaCl_2_, 1 mM MnCl_2_, 1 mM MgCl_2_, and 3 µg soybean agglutinin lectin peroxidase (specific for GalNAc) for 16 h at 20 °C. The membrane was then washed twice in PBS for 10 min before detecting and analysing the colonies using Immobilon™ chemiluminescent HRP substrate (Millipore) with ImageQuant™ RT ECL (GE Healthcare, Chicago, IL, USA), fitted with a 16-bit CCD camera.

### 2.3. Colony Analysis

Images were analysed using version 7 of the ImageQuant software (GE Healthcare, Chicago, IL, USA). Single colonies were selected and those in close proximity of another colony were disregarded to ensure minimal contamination resulting from two colony selection. Pixel intensity and area were calculated and corrected to remove background. Density measurements were tallied, the means plotted and compared to the control.

### 2.4. Bacterial Growth and Protein Expression

The starter inoculum of the bacterial strains were cultured overnight at 37 °C, 180 rpm. One-hundred-mL cultures of Luria Broth media were inoculated with 1 mL of the starter culture and incubated at 37 °C, 180 rpm. At an optical density (O.D) of 0.5 at 600 nm, 0.2% (*v*/*v*) L-arabinose was added to induce target protein expression through the araBAD promoter. No induction of the pgl pACYC (pgl2) plasmid was necessary due to constitutive expression. Cells were then left to incubate and express the protein for 4 h at the lower temperature of 30 °C 180 rpm in order to aid in the expression of correctly folded protein and minimise growth. The final OD of the cultures was measured and 40 OD units worth was harvested through centrifugation at 4 °C, 4500× *g* for 10 min. The supernatant was discarded and the pellet stored at −20 °C.

### 2.5. Periplasmic Protein Extraction

Protein pellets were thawed on ice and resuspended in 1 mL of periplasmic lysis buffer (20% sucrose, 1 g/L lysozyme, 30 mM Tris-HCl pH 8.5, 1× Halt protease inhibitor complex (Thermo Fisher Scientific, Massachusetts, MA, USA)) and extracted on ice for 2 h. Samples were centrifuged at 4500× *g* at 4 °C for 10 min. The supernatant, representing the soluble periplasmic fraction, was collected and the total protein quantity was measured using a Bradford protein assay.

### 2.6. SDS-PAGE and Western Blot Analysis

Five micrograms of periplasmic protein extract was added to 6 µL NuPAGE™ LDS sample loading buffer (4×) (Thermo Fisher Scientific, Massachusetts, MA, USA), 2.4 µL of sample reducing agent, with sufficient water added to make a final volume of 24 µL. Samples were heated to 80 °C for 10 min and left to cool to room temperature. Precast NuPAGE™ Novex 4–12% Bis-Tris gels (Thermo Fisher Scientific, Massachusetts, MA, USA) were loaded into the gel apparatus and the two chambers filled with the required MOPS SDS buffer. Samples were loaded into the wells and the gel run for 1.5 h at 180 V. The gel was extracted from the cast and washed in deionised water. Proteins were transferred to a nitrocellulose membrane using an iBlot^®^ (Thermo Fisher Scientific, Massachusetts, MA, USA). The membrane was subsequently blocked in blocking buffer (5% milk powder in Tris-Buffered Saline (TBS) with 0.1% *v*/*v* Tween20) for 1 h at room temperature. Three × 10 min washes of the membrane in TBS 0.1% Tween20 was followed by overnight incubation at 4 °C in blocking buffer with a His-tag antibody (Abcam^®^ Anti-6X His tag^®^ (HRP), Cambridge, UK) (1:10,000). The excess unbound antibody was washed off with 3 × 10 min washes in TBS 0.1% Tween20. The HRP linked antibody was detected on the blot using 10 mL of TMB-Ultra blotting solution (Thermo Fisher Scientific, Massachusetts, MA, USA) for 20 min. For the lectin peroxidase blot, the same procedure was followed except the antibody was replaced with soybean agglutinin (SBA) lectin (specific for GalNAc) peroxidase as described previously [10]. Pictures were captured using a 16-bit CCD camera. Images were analysed using Image Studio Lite v5.2. Bands were highlighted and the pixel intensity and area calculated to obtain the densitometry measurements with background correction factored into the analysis. Where applicable, a quantified standard of His-tag purified aglycosylated AcrA was used to measure the amounts of the target produced, as described previously [19]. Samples were run in triplicate and an unpaired t-test with Welch’s correction (assuming both the populations do not have the same standard error) was undertaken to look for statistically significant differences (P < 0.05). The quantification of total glycoprotein per litre of pgl2 IFN generated in flask culture was calculated through the analysis of the final OD of the culture and the amount of glycosylated target protein extracted from the periplasm of 30 OD units of culture as a percentage of the total protein present in the same 30 OD of periplasm. In brief, the final OD_600_ measurement of the culture was measured with 1 mL of OD_600_, 1.0 being equal to 1 OD unit. Thirty OD units were harvested, the periplasmic protein extracted, quantified and 5 µg loaded onto a gel for western alongside 0.5 µg of aglycosylated, His tagged pure AcrA standard. The target protein was quantified against the standard, and the amount of target protein present per 30 OD of periplasm was determined. This was then used to determine the total amount of target glycoprotein present in 1 L of culture.

## 3. Results and Discussion

### 3.1. MC4100 Cell Surface Representation of Glycans

Constructs were transformed into *E. coli* MC4100 along with the protein glycosylation machinery Pacyc (pgl2). As a positive control, the cell line MC4100 with pEC (acrA_MCS) pACYC (pgl2) was created. For the negative control, just the pEC (acrA_MCS) plasmid was transformed into the cell line MC4100. MC4100 was chosen because of the presence of the *waaL* gene. This gene expresses an *O*-antigen ligase that can recognise the glycan as a potential substrate and attach it to a lipid A core, which subsequently gets exported to the cell surface, placing the glycan on the extremity of the cell. By having this pathway intact and not inducing the expression of the target protein, it allows the presentation of the glycans on the cell surface. This enabled semi-quantitative analysis of glycan production through immunoblotting the surface of the cells with a lectin specific to the sugar residues in the pgl2 glycan.

**Figure 1:**
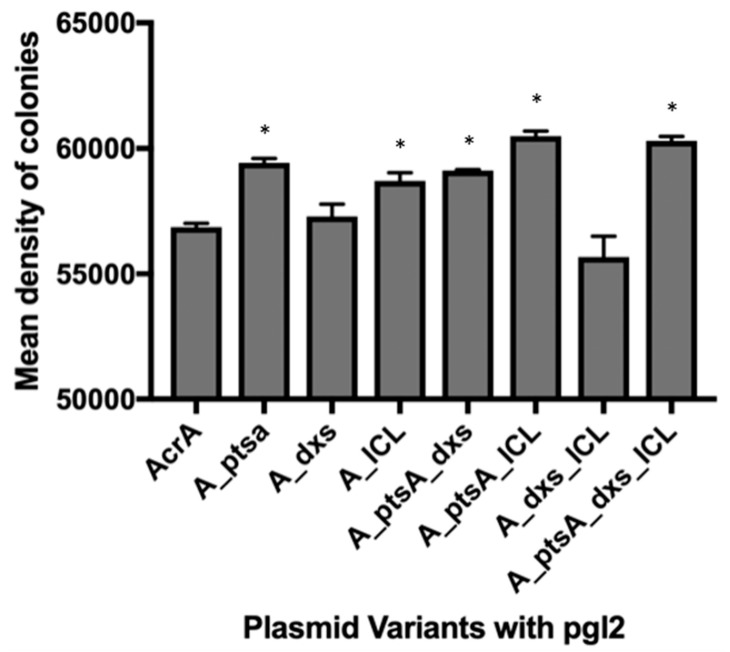
Mean density measurements for colonies in positive control strain (AcrA is *acrA* with *pgl2*) and engineered strains. Error bars represent the standard deviation of 55 colonies. Asterisks (*) above the bars indicate strains of significant difference from the control (Unpaired t-test with Welch’s correction, n = 3; P < 0.05).

Cell surface representation of glycans produced by the various cell lines are shown in Supplementary Files (Figure S1). Fifty-five single isolated colonies were analysed at random on each plate and their densitometry compared to that of the positive control. From this, the glycan production of each strain was measured and compared based on relative intensity (Figure 1). Although five of the engineered strains expressed statistically significantly more glycans on their cell surface, changes were very small. Of the combinations tested, the cells expressing both *ptsA* and *icl* had the biggest influence on glycan production, but they only had a 6.4% increase (Figure S1B). However, cells expressing *dxs* alone or in combination with *icl* had an inverse effect (Figure 1). The 3.2% increase observed with pEC (acrA_ICL) was reduced to a negative 2.1% change when *icl* was over expressed in combination with *dxs* in pEC (acrA_dxs_ICL). In subsequent experiments, a bacterial strain with a *waaL* deletion was used so that glycans were transferred to the expressed target protein. This enabled us to analyse whether the findings of the surface glycan screen were actually translated to overall glycoprotein production and glycosylation efficiency.

### 3.2. Measuring Glycoprotein Production Capability

#### 3.2.1. Expression of Glycosylated AcrA in E. coli Cells, CLM24

Constructs were transformed into *E. coli* CLM24, a strain that is *waaL* deficient, along with the protein glycosylation machinery pACYC (pgl2). As a positive control, the cell line CLM24 with pEC (acrA_MCS) pACYC (pgl2) was used. For the negative control, the pEC (acrA_MCS) plasmid was transformed into cells. The bacteria were grown in culture and induced with L-arabinose to express the target protein. SDS-PAGE and subsequent Western blotting was used to analyse production (using an anti-His antibody), with the densitometry of the bands used to calculate the relative glycoprotein production and glycosylation efficiency (Figure S2).

**Figure 2:**
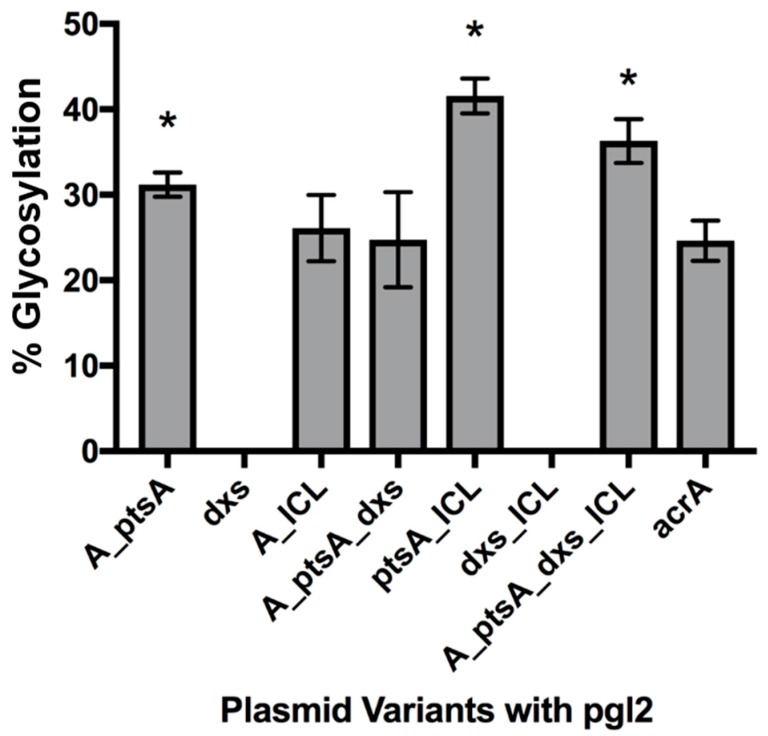
Glycosylation efficiency of the strains expressing *acrA*, the metabolic engineering genes and the pgl2 machinery. Cells without metabolic engineering of central carbon metabolism, CLM24 pEC(acrA) pACYC(pgl2), was included for a comparative control. Efficiency calculated through measuring the density of aglycosylated and glycosylated bands of AcrA. “A” denotes *acrA* expression and asterisks (*) above the bars indicate strains of significant difference from the control (Unpaired t-test with Welch’s correction, n = 3; P < 0.05). AcrA is the control strain comprising of the target protein gene (*acrA*) and glycosylation machinery contained in the pgl2 plasmid without any additional metabolic engineering elements.

Figure 2 shows the relative glycosylation efficiency of the engineered strains. Some are represented with an absence of data due to the lack of glycoprotein producing samples indicating a detrimental effect on glycan transfer to the protein target. Of the seven combinations tested, three were shown to have statistically different glycosylation efficiency when compared to the control. pEC (acrA_ptsA_ICL) gave the highest glycosylation efficiency, with a 1.69-fold increase. Two of the other significant strains, pEC (acrA_ptsA_dxs_ICL) and pEC (acrA_ptsA), caused a 1.48-fold and 1.27-fold increase in efficiency, respectively.

Two of the constructs were detrimental towards the glycosylation process within the cell, as no or few glycoprotein bands were observed on the Western blots. Both of the affected strains over expressed *dxs* (either alone or with *icl*). Overexpression of *dxs* reduces glycosylation efficiency when combined with the increased expression of both *ptsA* and *icl* (expressing all three *ptsA*, *dxs* and *icl* genes), compared to strains engineered with *ptsA* and *icl* alone. Of the seven combinations tested, the most efficient combination in terms of glycosylation efficiency was *ptsA* and *icL*, which raised efficiency from 24.6% to 41.6%. This 1.69-fold increase from the control was reduced to a 1.47-fold increase with the further overexpression of *dxs* and highlights its negative impact. The reduction of efficiency from 31.2% in pEC (acrA_ptsA) to 24.8% in pEC (acrA_ptsA_dxs) and the negative effect from *dxs* addition in pEC (acrA_dxs_ICL), whereby its inclusion inhibited the cells ability to produce detectable glycoproteins in two of the three biological replicates, adds to this argument.

The cell response to overexpression of *dxs* was unexpected, as this was previously shown to increase glycosylation efficiency 1.3-fold in a similar background strain, although with a different glycan structure [10]. Moreover, the growth rates of the strains tested did not differ significantly (data not shown). In our previous study, although overexpression of *dxs* showed a slight negative impact on the growth rate, this was not statistically significant. Interestingly, it did show a 1.6-fold reduction in recombinant protein yields [10]. DXP is used in isoprenoid biosynthesis, which is known to be involved in a wide variety of biological functions. It is therefore expected that fluxes within the isoprenoid metabolic network are tightly regulated, temporally and spatially. This makes it very difficult to dissect the mechanistic impact of DXP overexpression in terms of growth and protein production. When *dxs* was over expressed with a stronger promoter system (synthetic *trc* promoter), as in a previous study [29], growth yield (this was based on final OD) was severely inhibited, and it is hypothesised that this may be due to a drain on glycolytic intermediates necessary for balanced growth. It could be that if induced more moderately, growth is not impacted significantly, but when switched to protein production phase, the intermediates for protein production are affected.

In another study, it was elegantly demonstrated how promoter strength can affect the impact of *dxs* on downstream metabolic pathways in *E. coli* [30]. In the study, *dxs* was identified as the first enzymatic step in the production of a carotenoid, and in a wild type *E. coli* strain, increasing promoter strengths led to elevated carotenoid expression only to a certain level. It was hypothesised that other enzymes in the pathway become rate-limiting as well as cause toxic accumulation of the *dxs* product, DXP. In contrast, a strain engineered to overexpress these downstream enzymes showed a linear increase in carotenoid expression as promoter strengths for *dxs* were increased [30]. This implies that an *E. coli* glycoprotein platform strain would require enzymes downstream of *dxs* to be optimised to see the benefit of *dxs* overexpression. A map illustrating metabolic pathways downstream of *dxs* as well as the role of *icl* in central carbon metabolism is shown in Supplementary Files (Figure S3).

#### 3.2.2. Expression of Glycosylated IFNα2b in E. coli Cells, CLM24

**Figure 3:**
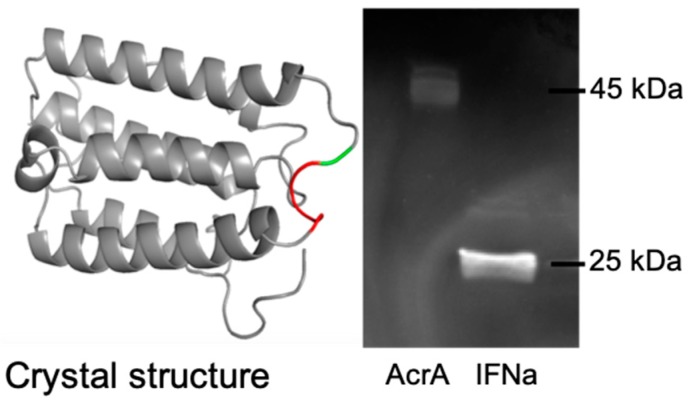
*N*-glycosylation of IFN-α2b in *E. coli* (left: crystal structure. GVGVT amino acid sequence was changed to EVNVT. In PDB, 1ITF GVGVT is at position 102–106. The T corresponds to the *O*-linked glycosylation site as residue T106 (The 102GVGV105 is marked in red and T106 in green); right: lectin peroxidase blot with AcrA control.

Three of the engineered cell lines are capable of improving the glycosylation efficiency of AcrA, and this is based on the hypothesis that the quantity of glycan precursors are limited during recombinant glycoprotein induction. Therefore, we expected that other glycoprotein targets should also have higher sugar occupancy levels in the engineered strains. We selected IFNα2b as this is an industrially-relevant, therapeutic protein. IFNα2b is a relatively small protein at 23.7 kDa. A single bacterial N-glycosylation consensus sequence of EVNVT was engineered from amino acid 102 to 106 (Figure 3). Human IFN fragments have been expressed in *E. coli* previously, including transport to the periplasmic space [31,32]. A Western blot using soyabean agglutinin lectin and lectin peroxidase was used to detect a glycosylated protein band to ensure the site was accessible for the *C. jejuni* oligosaccharyltransferase PglB (Figure 3).

**Figure 4:**
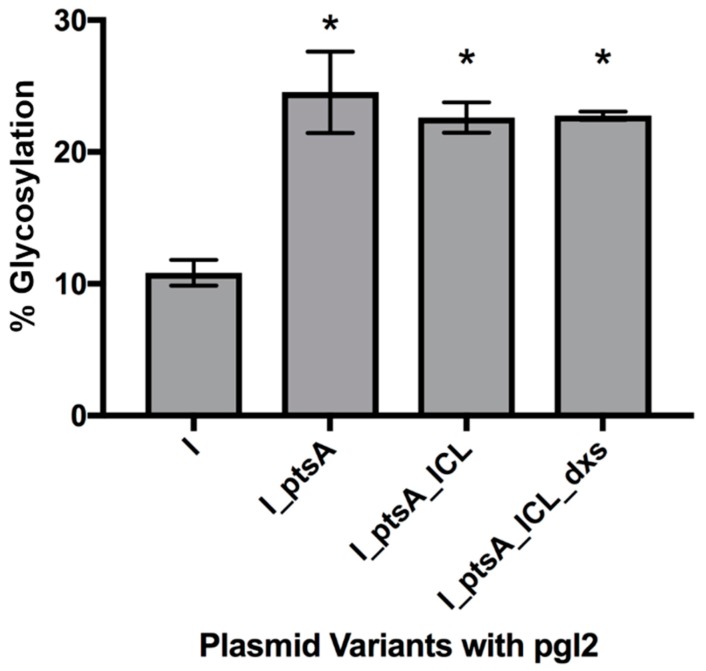
Glycosylation efficiency of the strains expressing *IFN-α2b*, the metabolic engineering genes and the pgl2 machinery. CLM24 pEC (*IFN-α2b*) pACYC (pgl2) is included for a comparative control. Efficiency was calculated through measuring the density of glycosylated and glycosylated bands of IFN-α2b. “I” denotes *IFN-α2b* expression and asterisks (*) above the bars indicate strains of significant difference from the control (Unpaired t-test with Welch’s correction, n = 3; P < 0.05).

As Figure 4 shows, the glycosylation efficiency of IFNα2b in control cells pEC (IFN_MCS) pACYC (pgl2) was approximately 10.9% (Western blots shown in Figure S4). However, all engineered cells showed statistically significant improvements in the ratio of protein which carried the hexasaccharide glycan structure. pEC (IFN_ptsA) was the highest with 24.5% glycosylated. pEC (IFN_ptsA_ICL) and pEC (IFN_ptsA_ICL_dxs) glycosylated with 22.6% and 22.8% efficiency, respectively.

**Figure 5:**
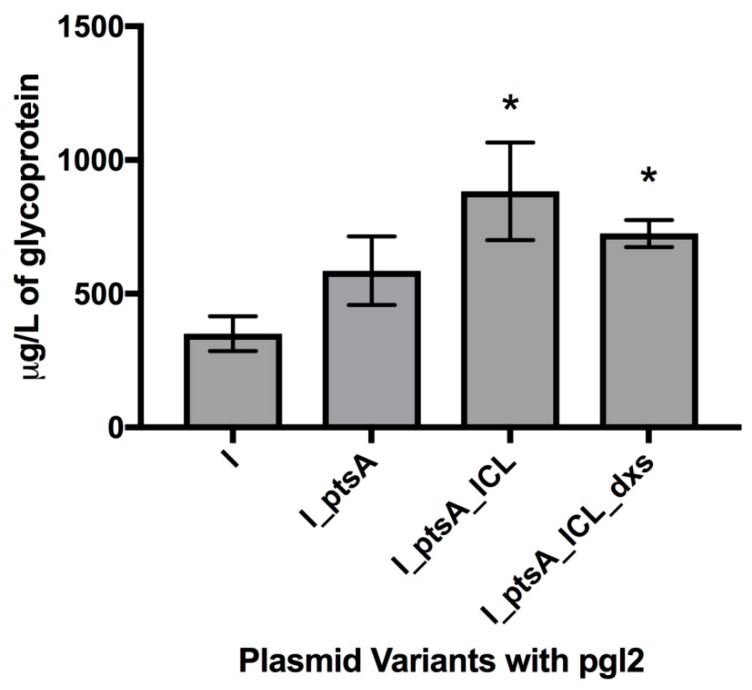
Average µg glycoprotein per litre of cell culture expressing *IFN-α2b* along with the metabolic engineering genes and the pgl2 machinery. CLM24 pEC (*IFN-α2b*) pACYC (pgl2) is included for a comparative control. Quantity was calculated through measuring the density of glycosylated bands of IFN-α2b compared to a loading control of 0.5 µg of purified AcrA. “I” denotes *IFN-α2b* expression and asterisks (*) above the bars indicate strains of significant difference from the control (Unpaired t-test with Welch’s correction, n = 3; P < 0.05).

We also quantified total IFN-α2b glycoprotein due to its industrial relevance, and pEC (IFN_ptsA_ICL) pACYC (pgl2) cells were the largest producer, with 882.9 μg L^−1^ compared to 351 μg L^−1^ in the control (Figure 5). The need to optimise the expression of *dxs* was again evident in these strains, but the successful improvement in *N*-glycosylation capability through overexpression of *ptsA* and *icl* was evident.

Variations in glycosylation efficiency in different proteins, for example, AcrA and IFN-α2b, are expected. These proteins are expressed to different levels and have very different structures, potentially affecting the accessibility of the PglB oligosaccharyltransferase. Moreover, AcrA has two bacterial *N*-glycosylation sites, N123 and N273, where the nitrogen on asparagine acts as the site for covalent attachment to the glycan, compared to just one site engineered into IFN-α2b at N104. Both the construction of glycans through glycosidic bonds and attachment of the hexasaccharide construct to polypeptide backbones requires energy in the form of ATP [33] and, therefore, less sites would reduce energy demands on the cell. However, both target proteins showed higher glycosylation efficiency when both *ptsA* and *icl* were over expressed. As suggested previously, the enhanced uptake and phosphorylation of sugars from the media is beneficial for an *E. coli* glycoprotein production strain, providing precursors for glycan construction. Moreover, using the strains constructed for this study, further conservation of carbon through increased flux through the glyoxylate pathway is a complementary modification to the cell. Unexpectedly, overexpression of *dxs* was detrimental here, even eliminating glycoprotein detection completely in some strains. Although it has been hypothesised that phenotypes related to increased *dxs* expression require similar optimisation of associated pathway enzymes, *dxs* is involved in isoprenoid production and therefore is used in diverse cellular processes beyond Und-P production. Although Und-P abundance could be a bottleneck in *N*-glycosylation, further studies would need to be undertaken to evidence this prior to identifying a gene target or tuning *dxs* expression that can work in tandem with *ptsA* and *icl* overexpression.

## 4. Conclusions

The physiology and genetics of *E. coli* are the most understood of all prokaryotic host cell factories for the production of recombinant proteins. Although the addition of glycans to proteins is not a native process in *E. coli*, there are advantages of incorporating this capability into the system, such as improving the stability of industrial enzymes through to diagnostic applications. Little is known about how the *Campylobacter jejuni N*-glycosylation system impacts on metabolism in *E. coli*, but here we aimed to combine gene modification targets from previous studies, based on the hypothesis that central carbon metabolism requires optimisation to improve glycosylation efficiency. Although combining the overexpression of genes involved in sugar uptake and phosphorylation with pathways that enable carbon conservation did not show large differences in glycan precursor production through a cell surface display screen, significant increases in glycosylation efficiency on recombinant proteins were revealed. AcrA and IFNα2b glycosylation increased 1.69-fold and 2.2-fold, respectively. However, not all modifications were positive (*dxs* overexpression), highlighting the need to test multiple cell engineering targets together and also the requirement for tuning the overexpression of individual genes for optimisations, providing a more comprehensive understanding of cellular constraints.

## Supplementary Materials

The following are available online at https://www.mdpi.com/2306-5354/6/1/27/s1, Figure S1: A: GalNAc specific lectin peroxidase screen against the pgl2 glycan represented by *E. coli* MC4100 cells containing pACYC(pgl2) and the various metabolic engineering plasmids. Target protein not induced. B: Graph showing the percentage change at the relative intensity of the colonies when compared to the control without the metabolic engineering genes. “A” denotes acrA expression and asterisks above the bars indicate strains of significant difference from the control (Unpaired t-test with Welch’s correction, n = 3; P < 0.05), Figure S2: A: Western blots of the seven constructs and the control expressing AcrA along with the pACYC (pgl2) machinery. His-tag antibody was used for detection of the target protein. The three bands for each strain represent the three biological replicates. B: A demonstration of Western blot binning for densitometry analysis. C: Raw densitometry data, Figure S3: Metabolic pathways for dxs (left) and icl (right), Figure S4: Western blots of control and engineered strains expressing IFN α2b along with the pACYC (pgl2) machinery. His-tag antibody was used for detection of the target protein. The three bands for each strain represent the three biological replicates. A: Lane 1: Novex protein marker, Lanes 2–4: IFN_pgl2, Lanes 5–7: IFN_ptsA_pgl2, Lane 8: 0.5 g AcrA. B: Lane 1: Novex protein marker, Lanes 2–4: IFN_ptsA_ICL_pgl2, Lanes 5–7: IFN_ptsA_ICL_dxs_pgl2, Lane 8: 0.5 g AcrA, Table S1: Sequence of the newly designed multiple cloning site and the primers used to amplify it, Table S2: The primers used to amplify the three genes of interest and the restriction sites used to insert them into pEC (acrA_MCS).
