# Supplementary material for: Engineering Pathways in Central Carbon Metabolism Help to Increase Glycan Production and Improve N-Type Glycosylation of Recombinant Proteins in E. coli
Source: Bioengineering (Basel). 2019 Mar 21;6(1):27. doi: 10.3390/bioengineering6010027 (PMC6466297; doi:10.3390/bioengineering6010027)
Supplement: Supplementary file 1 [file bioengineering-06-00027-s001.pdf]

Table S1. Sequence of the newly designed multiple cloning site and the primers used to amplify it.

Part	Sequence 5'- 3'
J23119	TTGACAGCTAGCTCAGTCCTAGGTATAATGCTAGC
MCS 5'	TGTCTTGACAGCTAGCTCAGTCCTAGTATAATGCTAGCAGCTCGCGGCCGCAGCTCCCATGGAGCCGGCCGGCCAGCTCTTAATTAAAGCTC
MCS 3'	GAGCTTTAATTAAGAGCTGGCCGGCCGGCTCCATGGGAGCTGCGGCCGCGAGCTGCTAGCATTATACCTAGGACTGAGCTAGCTGTCAAGACT
MCS Forward Primer	CCCGGGAGTCTTGACAGCTAGCTCAGTC
MCS Reverse Primer	GAATTCGAGCTTTAATTAAGAGCTGGCCG

Table S2. The primers used to amplify the three genes of interest and the restriction sites used to insert them into pEC(acrA_MCS).

Gene	Forward Primer	Reverse Primer	Restriction Sites
ptsA	GCGGCCGCAG GAGGTAAATA ATGGCCCTGA TTGTGGA	CCATGGTTACAGTTCCAG TTCATGTTGCAG	NotI, NcoI
dxS	CCATGGAGGA GGTA AATAATGAGT TTTGA TATTGCCAAA TACCC	GGCCGGCCTTATG CCAGCCAGGCC	NcoI, FseI
icL	GGCCGGCCAG GAG GTAAATAATG AAAAC CCGTACACAA CAAA	TTAATTAATTAGAACTG CGATTCTTCAGTG	FseI, PacI

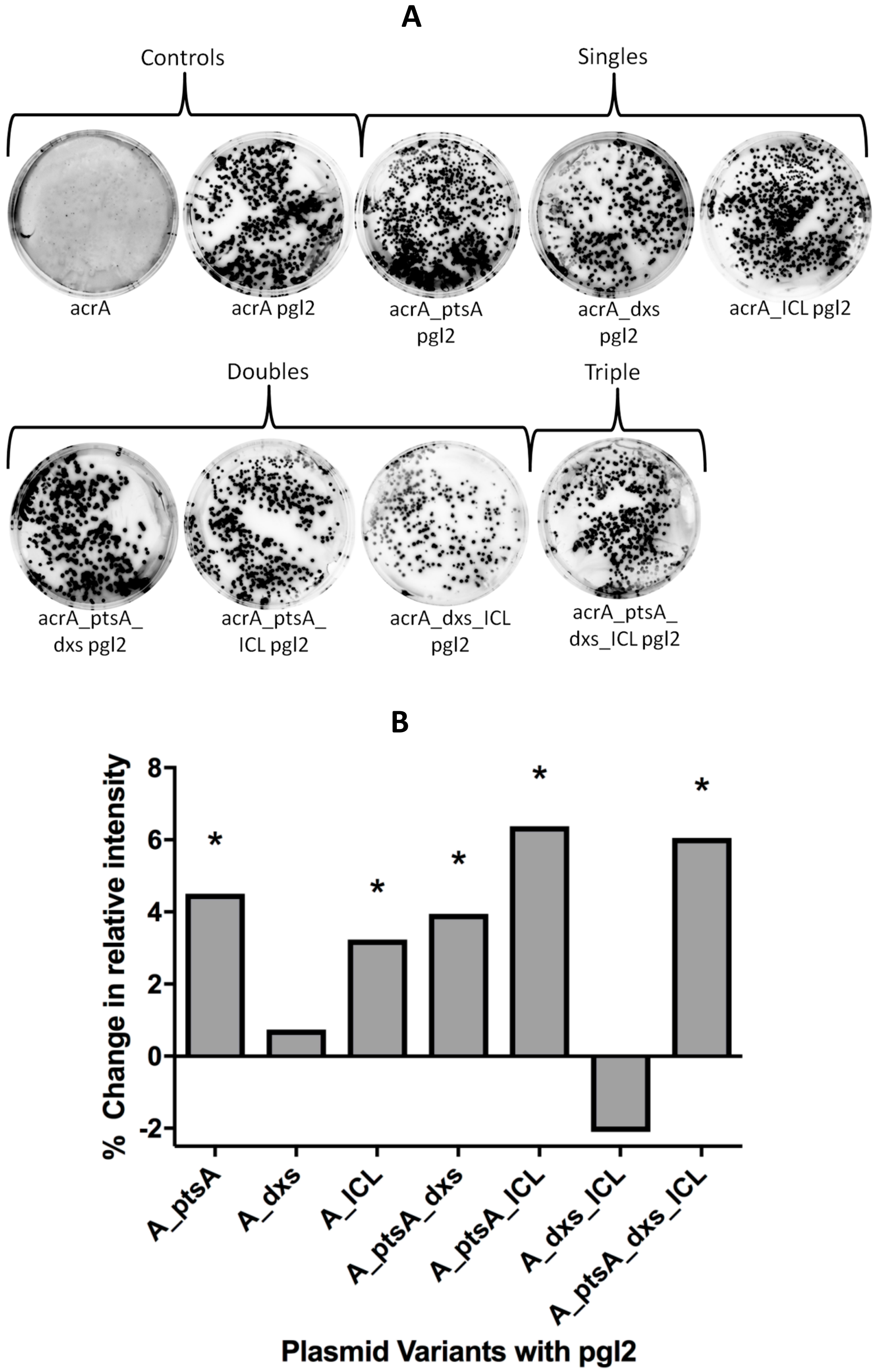

Figure S1. **A**: GalNAc specific lectin peroxidase screen against the pgl2 glycan represented by *E. coli* MC4100 cells containing pACYC(pgl2) and the various metabolic engineering plasmids. Target protein not induced. **B**: Graph showing the percentage change in the relative intensity of the colonies when compared to the control without the metabolic engineering genes. “A” denotes *acrA* expression and asterisks above the bars indicate strains of significant difference from the control (Unpaired t-test with Welch’s correction, n = 3; $P<0.05$).

A

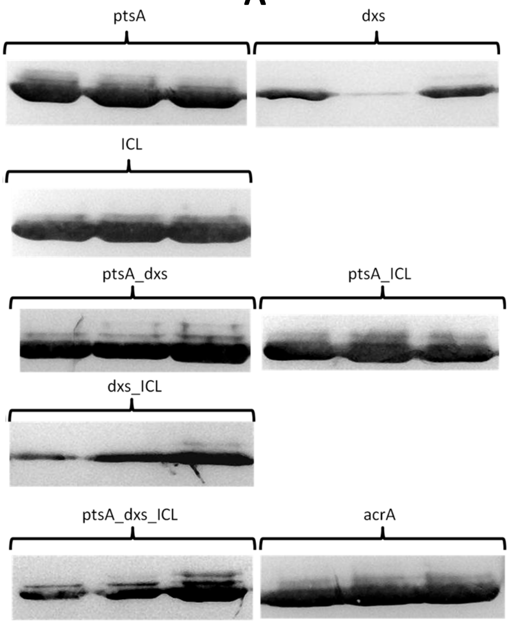

B

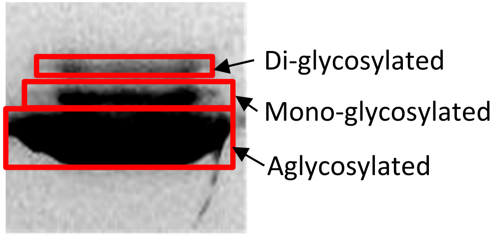

C

**ptsA**	1	2	3		Average	SD
aglyco	25886357	28647458	27562366		27365394	1391049
glyco	10864856	13431245	13019805		12438635	1378371
Total	36751213	42078703	40582171		39804029	2747666
efficiency	29.56326	31.91934	32.08257	average	31.18839	1.409772
				SD	1.409772	
**icl**	1	2	3			
aglyco	23990471	28561059	28063646		26871725	2507603
glyco	6698120	10628483	11683295		9669966	2627177
Total	30688591	39189543	39746941		36541692	5076590
efficiency	21.82609	27.12071	29.3942	average	26.11367	3.883256
				SD	3.883256	
**ptsA_dxs**	1	2	3			
aglyco	24285600	25688509	27120134		25698081	1417291
glyco	7350959	6468251	12125827		8648346	3043756
Total	31636559	32156759	39245961		34346427	4251086
efficiency	23.23565	20.11475	30.89701	average	24.74913	5.548176
				SD	5.548176	
**ptsA_icl**	1	2	3			
aglyco	13410269	14942704	13363165		13905380	898658.2
glyco	8643839	11219978	9922676		9928831	1288080
Total	22054109	26162682	23285842		23834211	2108465
efficiency	39.19378	42.88543	42.61249	average	41.5639	2.057113
				SD	2.057113	
**ptsA_dxs_icl**	1	2	3			
aglyco	21521764	28251804	38497994		29423854	8548589
glyco	11816660	14730774	24802644		17116693	6813839
Total	33338424	42982578	63300638		46540547	15294703
efficiency	35.44457	34.2715	39.1823	average	36.29945	2.564587
				SD	2.564587	
**Cont**	1	2	3			
aglyco	27405496	29290960	27722968		28139808	1009485
glyco	7768080	10671952	9288032		9242688	1452467
Total	35173576	39962912	37011000		37382496	2416183
efficiency	22.08499	26.70464	25.09533	average	24.62832	2.344967
				SD	2.344967	

Figure S2. A: Western blots of the 7 constructs and the control expressing AcrA along with the pACYC(pgl2) machinery. His-tag antibody was used for detection of the target protein. The three bands for each strain represent the three biological replicates. B: A demonstration of Western blot binning for densitometry analysis. C: Raw densitometry data.

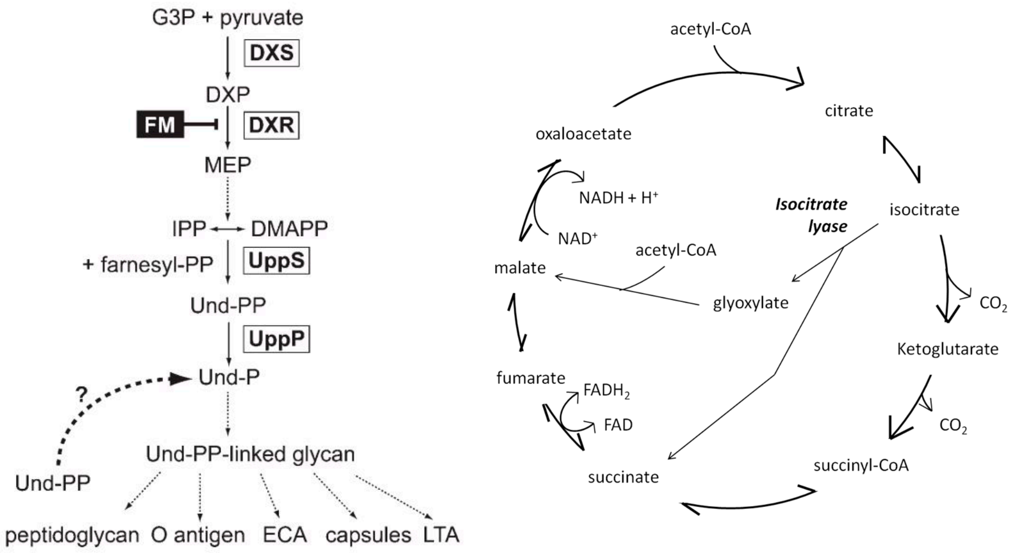

Figure S3. Metabolic pathways for *dxs* (left) and *icl* (right).

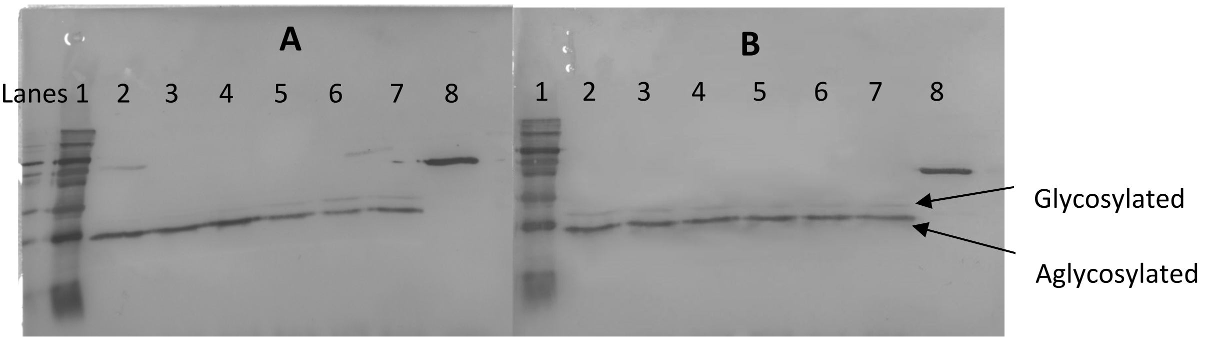

Figure S4. Western blots of control and engineered strains expressing IFN α2b along with the pACYC(pgl2) machinery. His-tag antibody was used for detection of the target protein. The three bands for each strain represent the three biological replicates. A: Lane 1: Novex protein marker, Lanes 2-4: IFN_pgl2, Lanes 5-7: IFN_ptsA_pgl2, Lane 8: 0.5 μg AcrA. B: Lane 1: Novex protein marker, Lanes 2-4: IFN_ptsA_ICL_pgl2, Lanes 5-7: IFN_ptsA_ICL_dxs_pgl2, Lane 8: 0.5 μg AcrA.
